# Fregene: Simulation of realistic sequence-level data in populations and ascertained samples

**DOI:** 10.1186/1471-2105-9-364

**Published:** 2008-09-08

**Authors:** Marc Chadeau-Hyam, Clive J Hoggart, Paul F O'Reilly, John C Whittaker, Maria De Iorio, David J Balding

**Affiliations:** 1 Department of Epidemiology and Public Health, Imperial College, St Mary's Campus, Norfolk Place, London, W2 1PG, UK; 2 Non-communicable Disease Epidemiology Unit, London School of Hygiene & Tropical Medicine, Keppel Street, London, WC1E 7HT, UK

## Abstract

**Background:**

FREGENE simulates sequence-level data over large genomic regions in large populations. Because, unlike coalescent simulators, it works forwards through time, it allows complex scenarios of selection, demography, and recombination to be modelled simultaneously. Detailed tracking of sites under selection is implemented in FREGENE and provides the opportunity to test theoretical predictions and gain new insights into mechanisms of selection. We describe here main functionalities of both FREGENE and SAMPLE, a companion program that can replicate association study datasets.

**Results:**

We report detailed analyses of six large simulated datasets that we have made publicly available. Three demographic scenarios are modelled: one panmictic, one substructured with migration, and one complex scenario that mimics the principle features of genetic variation in major worldwide human populations. For each scenario there is one neutral simulation, and one with a complex pattern of selection.

**Conclusion:**

FREGENE and the simulated datasets will be valuable for assessing the validity of models for selection, demography and population genetic parameters, as well as the efficacy of association studies. Its principle advantages are modelling flexibility and computational efficiency. It is open source and object-oriented. As such, it can be customised and the range of models extended.

## Background

FREGENE (FoRward Evolution of GENomic rEgions) is *C*^++ ^code for simulating sequence-level genetic data over large genomic regions in large populations. Unlike coalescent-based simulators it runs forward-in-time, which allows it to provide a wide range of scenarios for selection, recombination (crossovers and gene conversion), population size and structure, and migration. The advantages of forward simulators over coalescent simulators are discussed in [1]. A recent study [2] has shown that coalescent-based approaches can have serious limitations when there is a large recombination rate over the simulated genomic region. The main limitation of alternative forward-in-time simulators (FPG[3] and SIMUPOP [4]) is the size of genome and population that can be accommodated. A primary objective in the development of FREGENE has been computational efficiency. We implement a rescaling technique that greatly extends the feasible simulation size at the cost of some approximation. As a result, FREGENE can simulate a 20 Mb genome in 10 K diploid individuals over 300 K generations in a few days on a standard computer without rescaling, and only a few hours with rescaling.

Since its first publication [1], FREGENE has been extended to incorporate several new features. A general migration model has now been included. Selection at a locus can be switched off at a random time, and can be geographically restricted. The rescaling technique has also been fully automated. A new program, SAMPLE, has been developed that samples individuals from a FREGENE population, returning genotype and/or haplotype data. SAMPLE can also assign binary and/or continuous phenotypes generated under a user-specified model, and can replicate SNP and individual ascertainment schemes. Thus methods for the analysis of genetic association studies can be tested under realistic scenarios.

In this article we recap the main features of FREGENE, detail new developments, and illustrate its application by generating and analysing six large datasets. These datasets may serve as useful standards for testing methods to infer population genetic parameters, such as recombination rates and selection coefficients, and together with SAMPLE can be used to assess genetic association methods. We model three populations: two with constant population size (one panmictic and one with migration among three subpopulations) and one population that mimics the major features of worldwide human genetic variation [5]. The latter incorporates bottlenecks, periods of growth, and subdivision into three major continental groups. For each population there are two simulations, one neutral and the other adopting a complex selection model. Analyses of genetic diversity and the role of selection in these datasets are reported below.

FREGENE has been developed and tested under a Linux environment and uses the GNU scientific library (GSL). It can also be installed on a Mac platform (for installation details, see FREGENE documentation). Source code, executables, datasets and R scripts for generating figures such as those shown below are all freely available for download from , together with extensive documentation (see also Additional File 1).

## Implementation

### Overview

FREGENE simulates the evolution of a monoecious, diploid, potentially subdivided, population over non-overlapping generations. The genome consists of a single, linear chromosome, whose sequence is recorded as a list of sites at which the minor allele is present. Allele frequencies are checked at regular intervals, and if the minor allele has become the major allele at a site the sequence lists are appropriately updated. These updating operations are recorded such that it can always be determined whether the minor allele is ancestral or derived.

Parameters characterising the mutation, recombination, demographic and selection processes, are specified by the user, via either input files or the command line. A starting population is also specified, typically either a 'null' population with no diversity, or a population that is the result of a previous run of FREGENE. At the start of a FREGENE run, a *C*^++^ recombination object is invoked and uses the recombination parameters specified in the input file to stochastically assign recombination rates over the genome, including the locations and intensities of hotspots. The resulting 'recombination map' remains unchanged throughout the simulation.

Subsequently, each new generation is created one individual at a time. A parent is chosen at random, weighted by fitness, and a new sequence is generated by randomly recombining its two sequences. New mutations arise uniformly at random on the new sequence, regardless of current allelic status. Thus a mutation can arise on an already polymorphic site, in which case a 'double hit' mutation (affecting an ancestral allele) or 'back' mutation (derived allele reverts to the ancestral type) can occur, both of which are recorded. A second sequence is generated in the same way from a parent that is either the same as the first with probability equal to the selfing coefficient, or is a different individual chosen at random in the same way as the first parent. The pair of new sequences becomes an individual in the next generation.

### Recombination

**Figure 1:**
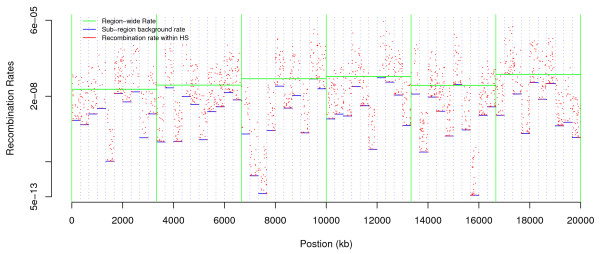
**Recombination rate (log scale) along the chromosome for populations A and B**. Solid green and dotted blue vertical lines represent first and last position of regions and subregions respectively.

The recombination model is based on a hierarchical approach similar to that of [5] (see Additional File 2). An example over a 20 Mb genomic region is shown in Figure 1. The model is highly flexible and incorporates uniform rate as a special case, as well as hotspots that can vary between regions in frequency and in intensity.

Although recombination rates are computed for each run, the random number seed used by the recombination module is recorded so that the recombination map can be maintained between successive runs.

### Demography

Currently, FREGENE accommodates constant population size, and growth/decline that is either exponential or instantaneous at the start of the run.

The population may be subdivided, in which case migration between subpopulations can occur according to arbitrary 'backward' migration rates: for an offspring in subpopulation *i*, its two parents are chosen from subpopulation *j *with probability *m*_*ij*_, which for *i *≠ *j *is specified by the user, and *m*_*ii *_= 1 - ∑_*j*≠*i*_*m*_*ij*_.

To allow successive runs of FREGENE where the output of one run becomes the input for the next, the main output file has the same structure as the input file: it contains the sequences in the final generation, and all the simulation parameters required as input. This facility allows complex demographic scenarios to be constructed from the simple demographic models offered within each FREGENE run. For example, in successive runs populations can split or subpopulations merge, and bottlenecks can be implemented in some subpopulations or the entire population.

### Selection

A key feature of FREGENE is that complex selection scenarios can be implemented: FREGENE allows for positive, negative and directional selection, as well as dominance and over-dominance at each site. The latter can lead to quasi-stable polymorphisms maintained under balancing selection. Crucially, the combined effects of multiple forms of selection at linked sites can be studied.

Novel mutant alleles are under selection with a specified probability. If so, two coefficients are assigned at random according to parameters set in an input file: *s*, which we call the 'selection' coefficient, and *h *the 'dominance' coefficient. The contribution to an individual's fitness at a site is *s *for a derived-allele homozygote, *sh *for a heterozygote, and zero for an ancestral-allele homozygote. Thus, for *s *> 0, a site is recessive if *h *= 0; co-dominant if 0 <*h *< 1; and over-dominant if *h *> 1. Individual fitness is one plus the sum of these contributions over all selected sites. After a derived allele reaches fixation it makes no contribution to fitness.

To model geographical variation in selection, due to differing environments, FREGENE allows subpopulation-specific selection. Each selected site is under selection either globally, or locally in the subpopulation where it arose, according to a probability specified by the user.

The selection coefficients of a site are constant over time until fixation, except that with a fixed probability at each generation selection is switched off, potentially reflecting a change in environment that eliminates the previous selective effect. The rate at which selection is switched off can be set to control the number of balancing polymorphisms at equilibrium.

Details of sites under selection (selection coefficients, generations when the selected mutant arose, and when fixed, lost, or switched off if any of these has occurred) are recorded.

### Scaling the population: Computation time and memory savings

A scaling technique, described in [1], can lead to dramatic reductions in computing time and memory use at the cost of some approximation. Scaling involves increasing all rate parameters (mutation, recombination, selection, migration) by a common factor *λ *> 1, while reducing by a factor of *λ *both the population size and the number of generations. FREGENE allows the user to specify *λ*, and it calculates the appropriate scaled parameters from the target values specified by the user. An undesirable feature of scaling is that the output population size is reduced by a factor of *λ*, but FREGENE now offers an option either to output this reduced population or to run additional generations during which scaling is relaxed and the population size expands linearly from *N*/*λ *to *N*.

### Generating genotype, haplotype and phenotype data: SAMPLE

The program SAMPLE samples individuals or chromosomes from FREGENE output to give, respectively, genotype or haplotype data. Association studies can be simulated by assigning continuous or binary (case and control) phenotypes to individuals according to a user-defined model. In the continuous case, the user specifies the phenotypic standard deviation, the number of causal SNPs (SNPs that affect the phenotype) and their heritabilities. In the binary case, the user specifies the prevalence of the trait, the number of cases and controls, the number of causal SNPs and their risk ratios. In either case the user can also supply a range for the allele frequency for each causal SNP. The SNP ascertainment scheme can be controlled by the user: the user can set the minimum minor allele frequency, or to simulate any given SNP ascertainment bias [6], a list of SNPs to be output can be specified by location of individual SNPs or ranges of SNP locations.

Ascertainment bias in structured populations, where cases and controls are sampled unequally in different subpopulations, is a potential problem in association analyses as it may result in false positives [7]. SAMPLE can simulate ascertainment bias, from a FREGENE subpopulation simulation, by allowing the user to specify the numbers of cases and controls from each subpopulation. This facility will be useful for testing methods that aim to correct for the effects of population structure.

## Results

### 'Ready to use' simulated data sets

Two standard population models, each with 10.5 K individuals, have been simulated over 20 Mb genomes, and the final generations are available as test datasets: population A is panmictic, while population B is subdivided into three subpopulations each of 3.5 K individuals. Migration rates in population B are all equal, and the common migration rate is chosen such that *F*_*ST*_, measuring the genetic distance between populations [8], is equal to 10%.

A third and more complex simulation (population C), over a 10 Mb genomic region, uses parameter values found by [5] to provide the neutral model that best fits the major features of current worldwide human genetic variation. This simulation used a per-site and per generation mutation rate of 1.5 × 10^-5 ^and required seven steps (note that all population expansions are instantaneous):

• **Founding population in Africa: **an homogeneous population (N = 25 K sequences) evolves for 125 K generations.

• **Expansion in Africa: **the population expands from 25 K to 48 K sequences and evolves during 17 K further generations.

• **Out of Africa (OoA) split and bottleneck: **among the 48 K sequences, 8.5% (= 4,080) leave Africa. Simultaneously, the population in Africa encounters a bottleneck of size ratio 0.8%, leaving 380 sequences in that subpopulation.

• **African and OoA expansion: **African population expands back to N = 48 K. Similarly the OoA population expands to N = 15.4 K and evolves for 3.5 K generations.

• **Asian and European split: **the OoA population encounters a bottleneck of size N = 1,360, and splits with N = 320 moving to Europe and N = 1,040 to Asia.

• **Asian and European expansion: **Asian and European populations both expand to N = 15.4 K, and evolve for 2 K generations. During this stage, migration occurs symmetrically, first between Asia and Africa (with rate 0.8 × 10^-5 ^per chromosome), and between Europe and Africa (with rate 3.2 × 10^-5 ^per chromosome).

• **Independent evolution of the three populations: **African, Asian and European populations evolve, without migration, during 200, 400 and 350 generations respectively, while each population expands to reach a final population size of N = 50 K sequences.

For each of the three populations described above, two simulated datasets are available, one neutral and one with selection. For all three populations, the same selection and recombination parameters were employed (Table 1), and the realised recombination rates were identical for the simulations of populations A and B (Figure 1). The selection model is intended to be illustrative rather than representing a realistic scenario for selection.

**Table 1:** Simulation parameters.

**General parameters **(only applies to populations A and B)	
Chromosome Length	20 *Mb*
# Generations	200, 000
# Sequences	21, 000
Per-site mutation rate	2.3 × 10^-8^
**Recombination model**	
Per site crossover rate:	1.1 × 10^-8^
Per site Gene conversion rate:	4.5 × 10^-9^
Proportion of recombination events occurring in hotspots	80%
Hotspot length:	2.0 *kb*
Gene conversion length:	0.5 *kb*
Mean distance between hotspots:	8.5 *kb*

**Selection parameters **(if applicable)	
Prop. of sites under selection:	5 × 10^-4^
Proportion of selected sites locally under selection:	0.5
Mean # generations before selected sites are switched off	50, 000
selection coefficient:	*s *~ 0.1 × N(0.005, 0.05^2^) + 0.9 × N(-0.01, 0.005^2^)
dominance coefficient:	*h *~ 0.8 × N(0.5, 0.2) + 0.3 × N(1.2, 0.2^2^)

**Figure 2:**
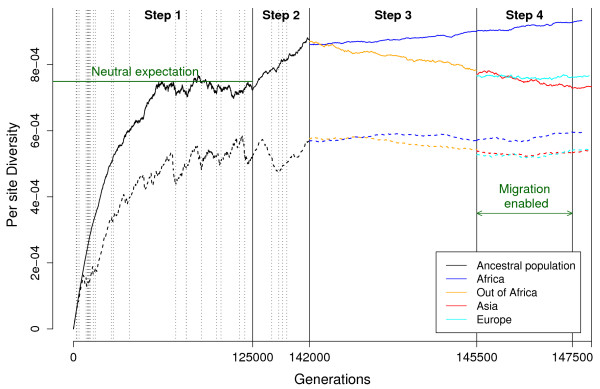
**Evolution of the per-site diversity for the worldwide human simulation (population C)**. Solid lines: neutral model; dashed lines: model with selection. Vertical dotted lines apply to the selection model and indicate when a strongly selected site (*s *> 0.075) went to fixation. Note that, for visual clarity, the time axis is scaled differently for different steps of the simulation.

The first section only applies to populations A and B; the corresponding values for population C are detailed in the main the text and some are also illustrated in Figure 2.

Results available for download include the main FREGENE output files, from which genotype, haplotype and phenotype data can be generated using SAMPLE. It is possible to generate haplotype data for subsequences with length defined by the user up to the full simulation length (20 Mb for populations A and B, 10 Mb for population C). In addition, the datasets available also include most of the optional FREGENE outputs to allow various analyses and plots.

### Genetic diversity in the worldwide human simulation (population C)

Figure 2 shows the evolution, for both neutral and selected simulations in Population C, of the allelic diversity, defined as the probability that two chromosomes chosen randomly within a given subpopulation carry different alleles at a randomly chosen site [9], and calculated as the average over sites of 2*f*(1-*f*), where *f *is the minor allele fraction. Allelic diversity equals the expected heterozygozity in the subpopulation under random mating.

During the first 125 K generations (step 1), diversity increases to reach an equilibrium value, which for the neutral model is close to the expected value under neutrality (horizontal green line), given by *E*_*h *_= 2*Nμ*/(1+2*Nμ*), where *μ *denotes the mutation rate per site and per generation. For the selection model, the diversity approaches an equilibrium value that is lower than *E*_*h*_. During the next 17 K generations (step 2), diversity under neutrality increases towards the new equilibrium expected value (*E*_*h *_≈ 1.4 × 10^-3^) but does not reach it, whereas under selection diversity is little affected by the population expansion. During steps 1 and 2, when a strongly selected site (*s *> 0.075) reaches fixation (vertical dotted lines), a trough in diversity is typically observed, corresponding to the effect of a selective sweep.

After the OoA split (generation 142 K), diversity slightly drops in the African population, due to the bottleneck, and then increases almost linearly until the end of the simulation. Diversity for the OoA population decreases linearly during step 3, approaching the equilibrium value of 7.1 × 10^-4^. The Asian and European populations have the same size (N = 15.4 K) during step 5, but diversity in Europeans is slightly higher than in Asians, due to a fourfold higher migration rate between Africa (with higher diversity) and Europe than between Africa and Asia. Trends highlighted for the neutral model also generally apply to the selection simulation, but with reduced diversity due primarily to the effect of selective sweeps in reducing diversity.

### Assessing the effect of selection: analysis of populations A and B

Table 2 summarizes diversity according to both demography (population A or B) and neutrality/selection. Under our modelling assumptions, selection impacts the evolution of diversity much more strongly than does demography, relative to the diversity in the neutral panmictic simulation. Selection reduces diversity substantially under panmixia, and less so in the subdivided population, in part because at 50% of selected sites the effect is local to the subpopulation. For neutral simulations, subdivision has a modest effect in increasing diversity within subpopulations and overall, compared with the panmictic population. Migration rates were set to ensure *F*_*ST *_= 10%, which permits sufficient mixing such that the overall diversity remains close to the within sub-population diversity, both for the neutral and selection simulations.

**Table 2:** Mean diversity for populations A and B.

	**Population A (panmictic)**		**Population B (subdivided)**		
	Diversity		Overall Diversity		Within-subpopulation Diversity
**Neutral**	9.33 × 10^-4^	(206 K)	9.82 × 10^-4^	(206 K)	9.58 × 10^-4^
**Selection**	6.72 × 10^-4^	(180 K)	8.06× 10^-4^	(198 K)	7.82 × 10^-4^

Mean diversity over the final 50 k generations (number of polymorphic sites), for populations A and B under the neutral and the selection scenarios.

**Figure 3:**
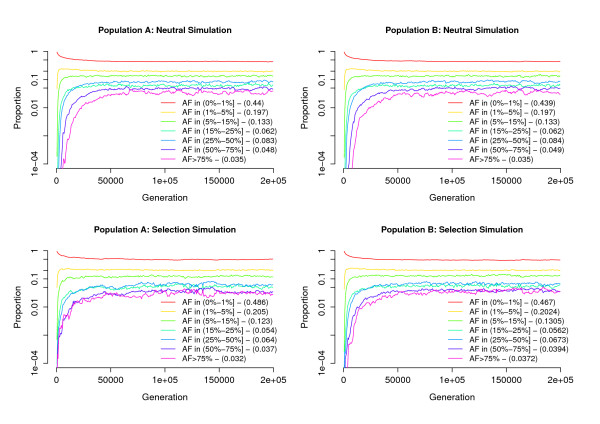
**Evolution of the distribution of allele frequencies**. Populations A (left) and B (right), simulated without (top) and with (bottom) selection. The mean proportion of sites within each allele frequency range, averaged over the final 100 k generations, is shown in parentheses.

Figure 3 presents the distribution of ancestral allele frequency (AAF) at polymorphic sites in populations A and B. For the neutral simulation, this distribution is very similar for the two populations. At equilibrium, around 40% of sites have AAF < 1%, while 20% have 1% < AAF < 5%, and 13% have 5% < AAF < 15%. Because the simulation starts with no variation, it takes longer for the proportion of sites with high AAF to stabilise: more than 60 K generations for the proportion of sites with AAF > 75% to become stable, compared with around 10 K generations for sites with AAF < 15%.

Under selection, there is a greater proportion of polymorphic sites with low AAF: for population A, 49% of polymorphic sites have AAF < 1%, compared with 44% in the neutral model. The impact of selection on the distribution of allele frequencies differs between panmictic (A) and subdivided (B) populations: sites with AAF < 5% are slightly less frequent in the subdivided population (67% vs. 69%), while sites with AAF > 50% are slightly over-represented (7.7% vs. 6.9%). The number of selected sites that went to fixation is 261 in population A, compared with only 146 in population B.

**Figure 4:**
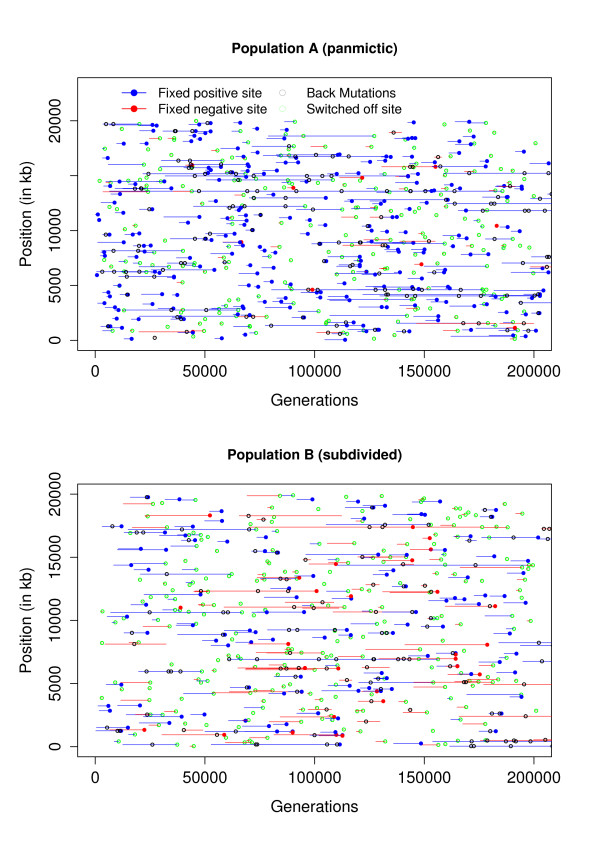
**Selected sites map**. Lines indicate the life-spans of sites under selection that reached fixation for the derived allele in populations A (top) and B (bottom). Red and blue circles indicate time of fixation of, respectively, positively (*s *> 0) and negatively (*s *< 0) selected sites. Also shown are selected sites at which selection was switched off (green), and at which a back mutation occurred (black).

The rate of fixation of selected sites is reduced by subpopulation structure, and also because in our simulations of population B half of the selected sites are neutral outside the subpopulation in which they arose. Thus, to reach fixation, a positively selected site will have to migrate out of the subpopulation in which it arose into every other subpopulation, where it may not be under selection. Figure 4 shows the life-spans of selected sites and gives further clues to interpret the differential effect of selection in panmictic and subdivided populations.

The probability of a double hit mutation is proportional to the number of polymorphic sites, and hence these are more common in population B (1.35 M vs. 1.26 M in population A, results not shown). The probability of a back mutation on a given site is an increasing function of the allele frequency on that site, and thus there were fewer back mutations in population A (117 K) than in population B (148 K). In Figure 4, we only represented back mutations occurring on selected sites (148 for population A, and 123 for population B). In both populations, back mutations mainly occur on sites that remain for a long time in the population, and thus often arise at balancing sites.

**Figure 5:**
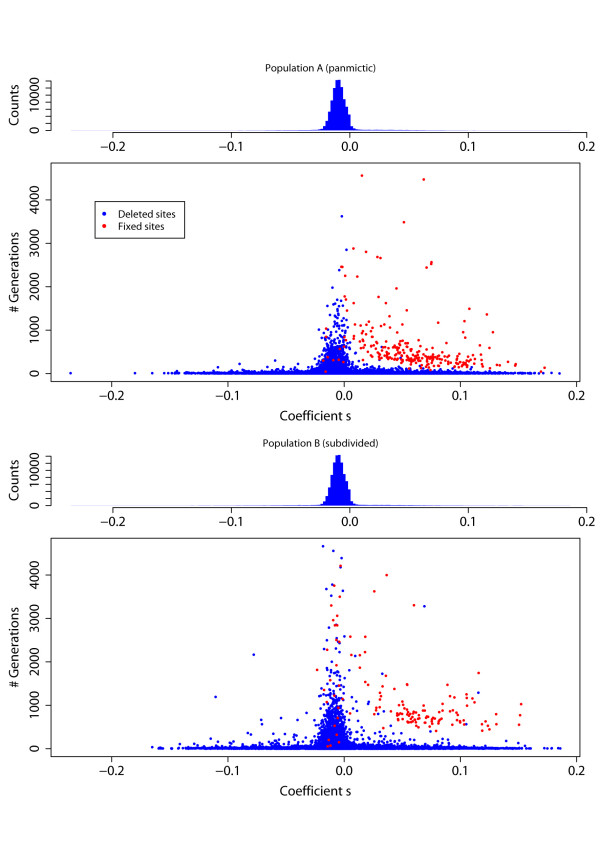
**Distribution of selection coefficients and time under selection**. The scatter plots show the *s *selection coefficient (*x*-axis) and the total time that the site remained polymorphic (*y*-axis), for all selected sites in populations A (top) and B (bottom). Red and blue indicates sites at which the derived allele reached fixation or was lost, respectively. The histograms show the distributions of *s*.

The impact of selection with or without subpopulation structure is summarized in Figure 5, which represents the time selected sites remain polymorphic as a function of the selection coefficient *s*. As discussed above, this time is greater in a subdivided population, regardless of *s *value. For positively selected alleles that reach fixation, the time required tends to reduce in both mean and variance with increasing *s*. As expected, the vast majority of selected sites at which the derived allele is lost had *s *< 0, and the smaller *s *is the quicker the loss, but derived alleles with *s *< 0 do sometimes reach fixation. Interestingly, time to fixation seems not to depend on the value of *s *for *s *< 0. Detailed tracking of the most negatively selected sites reaching fixation reveals that most were within 50 kb of a positively selected site that went to fixation at about the same time. Thus hitchhiking appears to be responsible in large part for the fixation of sites with *s *< 0. In population B, 29 such sites went to fixation, compared with only 11 in population A, reflecting the enhanced opportunities for hitchhiking created by the longer life-span of positively-selected sites in a subdivided population.

## Conclusion

FREGENE incorporates many useful features for population biologists and genetic epidemiologists, and has already been used to assess methods for the analysis of genome-wide association studies [10-12]. An important feature that overcomes a limitation of other software for simulation under selection is that the combined effects of different forms of selection – adaptive, purifying and balancing – can be studied in a single FREGENE run. Its flexibility could be improved further: for instance, users can define their own recombination model by altering the existing *C*^++ ^object. Moreover, structural variants, such as genomic inversions and copy number polymorphisms, could be incorporated into the model with further work. In contrast, accommodating structural variation within coalescent-based simulators presents a distinct challenge. Individual variability in the recombination map could also be considered: for instance, each individual could have their own recombination map, with hotspot intensity/presence dependent on sequence-content for example, which is transmitted to their offspring with minor changes. FREGENE and SAMPLE are open source and as such users are free to contribute additional features, or make any other improvements.

## Availability and requirements

• Project name: FREGENE

• Project home page: 

• Operating system(s): Linux/Unix – Mac

• Programming language: *C*^++^

• Other requirements: GNU Scientific Library (GSL)

• License: GNU GPL

• Any restrictions to use by non-academics: none

## Authors' contributions

MCH contributed to the FREGENE code, including many of the new developments described here. He generated the datasets and the figures and led on the drafting of the paper. CH contributed to the FREGENE code, wrote the SAMPLE code and contributed to the writing of the manuscript. POR, JW and MDI gave biological, statistical and computational advice to the project and commented on the manuscript. DB led the project and edited the manuscript. All authors have read and approved the final manuscript.

## Supplementary Material

Additional file 1 **FREGENE and SAMPLE codes**. this file contains FREGENE and SAMPLE source codes, together with the extensive documentation, and example files to run FREGENE. Click here for file

Additional file 2 **FREGENE recombination model in detail**. this text describes in greater details the recombination model implemented in FREGENE. Click here for file
