# Supplementary material for: Fregene: Simulation of realistic sequence-level data in populations and ascertained samples
Source: BMC Bioinformatics. 2008 Sep 8;9:364. doi: 10.1186/1471-2105-9-364 (PMC2542380; doi:10.1186/1471-2105-9-364)
Supplement: Additional file 1 — FREGENE and SAMPLE codes. this file contains FREGENE and SAMPLE source codes, together with the extensive documentation, and example files to run FREGENE. [file 1471-2105-9-364-S1.tgz › Fregene/documentation.pdf]

FREGENE: Sequence-level simulations over large genomic regions in large populations

DOCUMENTATION

August 11, 2008

Overview

FREGENE is a C++ program that simulates sequence-like data over large genomic regions in large diploid populations. Unlike coalescent-based simulation tools, such as MS (Hudson, 2002), FREGENE works forwards-in-time which allows a wide range of demographic and selection scenarios to be implemented. Many such models are already incorporated into FREGENE, and since it is open source users can modify or extend these. Coalescent methods have difficulty incorporating large amounts of gene conversion or crossover (Hoggart *et al.* 2007), whereas these pose no particular problem for FREGENE. FREGENE offers a flexible model for recombination hotspots, and can readily simulate regions up to tens of Mb on a standard desktop computer.

The principle limitation of forward-in-time algorithms is computational, since the entire population must be tracked through time, not only the chromosomes that are ancestral to the observed sample. FREGENE implements many features to enhance computational efficiency, and includes a rescaling option that greatly reduces computation time at the cost of some approximation.

The program SAMPLE, that comes with the FREGENE package, generates samples of individuals from a FREGENE output population, together with a phenotype that depends on the genotype at one or more SNPs and may be binary (case/control) or Gaussian. SAMPLE can also summarize the SNP minor allele frequency (MAF) spectrum and calculate r^2 values for SNP pairs.

For further details about FREGENE, including the rescaling, see:

> Chadeau-Hyam M, Hoggart CJ, O'Reilly PF, Whittaker JC, De Iorio M, Balding DJ (2008). FREGENE: Simulation of realistic sequence-level data in populations and ascertained samples . *BMC Bioinformatics* **in press**.
>
> Hoggart CJ, Chadeau-Hyam M, Clark TG, Lampariello R, Whittaker JC, De Iorio

M, Balding DJ (2007). Sequence-level population simulations overvlarge genomic regions. *Genetics* **177**: 1725-1731, 2007, doi: 10.1534/genetics.106.069088.

Please cite these articles in any publication that uses FREGENE.

FREGENE is free to use, distribute and modify, under the terms of the GNU General Public License as published by the Free Software Foundation; either version 3 of the License, or any later version. In particular FREGENE comes WITHOUT ANY WARRANTY.

Please report any problems or bugs to d.balding@ic.ac.uk

Summary of FREGENE's modelling assumptions

FREGENE simulates a, possibly subdivided, population of monoecious, diploid, individuals whose genomes consist of a single, linear chromosome. The population evolves over non-overlapping generations according to a Wright-Fisher model, with or without selection.

- **Mutation**: a two-allele, symmetric mutation model: multiple mutations at a site are allowed; the mutation rate is the same for each allele at each site.
- **Recombination**: both crossovers and gene conversions, with rates that may be uniform or vary along the chromosome at both broad and fine scales (the latter corresponding to hotspots).
- **Population size**: constant, or exponential growth.
- **Migration:** within a subdivided population; the islands can have different population sizes.
- **Selection:** A pre-specified proportion of sites is non-neutral. At sites under selection, fitnesses are allocated to each genotype stochastically, according to distributions that can allow directional (positive or negative) and balancing selection scenarios. Selection operates additively over sites. In a divided population, it can be local to the subpopulation in which the selected mutant arose, or global.

Many of these assumptions are easy to relax by small changes to the source code, but typically at a cost in computational efficiency.

Features of the implementation

- For computational efficiency, each chromosome is represented as a list of sites at which the minor allele is present
  - If a derived allele becomes the major allele, then the allele is “swapped” so that the ancestral allele is now recorded.

    – FREGENE outputs the "swap" status of each site, *i.e.* whether the minor allele is derived or ancestral.

- The main FREGENE output file, in xml format, summarizes the parameters of the simulation and includes the chromosomes present in the final generation. It is formatted so that it can be re-processed as an input file, which allows complex demographic scenarios to be built up from simple components, via repeat calls of FREGENE.

- FREGENE requires about 2 days on a standard desktop computer to simulate the evolution of a 10 Mb chromosome in 10K individuals over 100K generations. However, by **rescaling** the number of generations, population size, and the rate parameters, the computing time can be greatly reduced at the cost of some approximation. For example, with ten-fold rescaling the above simulation time is reduced by a factor of 64 to less than 1 hour. Disadvantages of rescaling include:

    1. Reduced population size; however, by setting a switch the user can ask FREGENE to run extra generations in which the rescaling is relaxed, bringing the population size up to its desired level.
    2. More mutations arising at sites that are already polymorphic ("double hit", or "back" mutations); FREGENE tracks these so this effect can be monitored.

See Hoggart *et al.* (2007) for further details.

User Guidelines

This documentation details options and features implemented in FREGENE. For a quicker description of its basic functionalities please refer to the 'quick start guide' at the end of this document.

1 Installation of FREGENE

1. FREGENE is a `C++` program that uses packages from the GNU Scientific Library (GSL), available at:

    `ftp://ftp.gnu.org/gnu/gsl/`

    For Linux users, the installation is straightforward and documented in the downloaded archive.

    For Mac users we suggest to:

- Download and install MacPorts, from

  `http://www.macports.org/`,

- Then type:

  `sudo port install gsl`

- Change the library path in the command line (or in .profile file, in the home directory, to get the correct path exported at each boot):

  ```
  export PATH=$PATH:/opt/local/bin:~/local/bin
  export LIBRARY_PATH=$LIBRARY_PATH:/opt/local/lib
  export CPATH=$CPATH:/opt/local/include
  ```

FREGENE has been developed and tested using the `g++` compiler on several Linux platforms. Compilation using alternative compiler may require some modifications in the `makefile` file.

2. Download `fregene.tgz` and unpack it by typing `tar -xzvf fregene.tgz` to generate the `Fregene` directory.

3. Type `cd Fregene/Fregene_source` and then `make` to create the executable file `fregene`.

4. To use SAMPLE, type `cd ../Sample` and type `make` to create the executable file `sample`.

5. To check the installation, and create the example files mentioned below, move to the `Example` directory and run the example script by typing `./fregene_example.sh` and then `./fregene_sample.sh`

2 Contents of the FREGENE Package

2.1 `Fregene_source` directory

This directory contains the FREGENE source code. The main program is `fregene.cc` and all other files are routines that are called by the main program. In each file, a variable called `DEBUG` is defined and set to 0 by default. If the user changes it to 1 and recompiles the program then step-by-step details of the program will be printed out on the standard output device (or log file). This option is useful to understand how FREGENE works, but it usually generates very large log files.

2.2 `Sample` directory

This directory contains the SAMPLE source code. The main program is `sample.cc` and all other files are routines that are called by this program.

2.3 `Common` directory

It contains:

- Routines to read/write xml files.
- GSL-based routines to enable random number generation.

2.4 `Example` directory

This directory illustrates how to use FREGENE. It provides one example of each input file required by FREGENE as well as a shell file to run FREGENE.

3 Running FREGENE

FREGENE can be run in a terminal or using a shell file. The minimal command has the form

```
fregene -i infile1 -p infile2 -recomb infile3 -o outfile
```

Input parameters are specified in the three input files, -i, -p, and -recomb, but some can also be set via additional command line options (see Section 5). However, the input files specified by the `-i`, `-p`, and `-recomb` arguments are always required and can be used to set all parameters. If a variable is assigned a value in both the command line and an input file, the latter setting prevails. However, if the -SELECT command line option is not set, any selection-related parameters in the input files will be ignored. Similarly for the -mg option and any parameters related to population subdivision.

FREGENE can readily be used without studying all the options in this document, by modifying the files specified by the `-i`, `-p`, and `-recomb` arguments of `Example/fregene_example.sh`.

3.1 Input Files

Initial population (`-i file_name`; required)

This xml-format input file details the chromosomes in the starting population. Most often the initial population will either be

- invariant (all chromosomes the same), or
- the final generation of a previous run of FREGENE.

**Variable Name**	*(Default, if applicable)*	**Description**
$< IS\_SCALED >$	*(1)*	1 if the output population is scaled, 0 if unscaled
$< SCALING\_FACTOR >$	*(1)*	Specifies the scaling factor (real ≥ 1)
$< GROUPS >$	*(1)*	Number of subpopulations (integer ≥ 1)
$< GROUPS\_SIZE >$		Sizes of the subpopulations (# chromosomes; one even integer for each subpopulation, separated by spaces)
$< SEED >$	*(1)*	Seed for the random number generator (integer)
$< NB\_SWAPPED\_SITES >$	*(0)*	Number of swapped sites (*i.e.* sites at which the minor allele is ancestral)
$< LIST\_SWAPPED\_SITES >$		Positions of all "swapped" sites
$< NB\_SELECTED\_SITES >$	*(0)*	Number of sites under selection
$< POSN\_SELECTED\_SITES >$		Positions of sites under selection
$< SEL\_GENERATION >$	*(0)*	The generations when each selected allele arose
$< SEL\_COEF >$		The selection coefficient s of selected sites
$< SEL\_DOM >$		The dominance coefficient h of selected sites
$< GROUP\_SELECTED\_SITES >$		The subpopulation(s) in which the site is under selection if =100, the site is globally under selection

Table 1: Optional tags for the `-i` input file. These are written to the `-o` output file and hence are always set if the output file is used as input for a subsequent run of FREGENE. Variables are described in further detail in Section 4.2.

The `-o` output file is structured so that it can immediately become the `-i` input file for a subsequent run of FREGENE.

The following tags are **required** and do not have a default value:

- $< SEGMENTS >$: The initial number of chromosomes in the population. Should be a positive, even integer (= twice the number of diploid individuals).

- $< MAX\_MUT\_CHROMO >$: The maximum number of minor alleles on any one chromosome in the initial population; zero for an invariant starting population.

- $< MAX\_MATRIX\_SIZE >$: The maximum number of elements of the matrix specifying the chromosomes. This value is platform-dependent: if too large for the computer's RAM, FREGENE may not run. Default is 7×10^7 (approximately corresponding to 300 Megabytes at 4 bytes per integer).

- $< CHROMO\_LENGTH >$: The chromosome length (in Mb).

- $< DATA >$: Describes the genetic variation in the initial population. An invariant population is represented by a single line containing only `0` (as in `Example/data/in_example.xml`). Otherwise, each line represents a chromosome, and lists the positions of sites carrying the minor allele, terminated by a `0`. The lines are ordered so that chromosomes $2k-1$ and $2k$ represent individual k.

The **optional tags**, mainly related to simulation and output options, are briefly described in Table 1. Some of these can also be set in the command line (see Section 5).

See `Example/data/in_example.xml` for an example with an invariant starting population, and `Example/data/rin_example.xml` for an example in which the starting population has been generated by a previous FREGENE run.

3.1.1 Evolutionary and simulation parameters (`-p file_name`; required)

Simulation and mutation parameters		
**Variable Name**	*(Default, if applicable)*	**Description**
< NO_GENER >	*(0)*	Length of the simulation run in generations.
< DELETION_INTERVAL >	*(0)*	# of generations between deletion operations.<br>Deletion operations check each site for alleles that have gone to fixation or vanished, or for a minor allele that has become the major allele or vice-versa.<br>Homozygozity is also computed.<br>Default value of 0 means these operations are performed every generation, which carries a computational overhead.
< MIGRATION >	*(I)*	Matrix giving backward migration rates between subpopulations.<br>Ignored if `-mg` option is not in command line.
< MUTAT_RATE >	(2.5×10^{-8})	Mutation rate (/site/generation)
Parameters of the selection model **(`-SELECT` *must be set in command line)***		
**Variable Name**	*(All default to 0)*	**Description**
< PROP_SEL >		Proportion of new alleles that are non-neutral
< SEL_COEF_POS >		Mean for the positive distribution of s
< SEL_COEF_SD_POS >		SD for the positive distribution of s
< SEL_COEF_NEG >		Mean for the negative distribution of s
< SEL_COEF_SD_NEG >		SD for the negative distribution of s
< PROP_POS_SEL_COEF >		Mixture weight of the positive distribution of s
< SEL_DOM_POS >		Mean for the positive distribution of h
< SEL_DOM_SD_POS >		SD for the positive distribution of h
< SEL_DOM_NEG >		Mean for the negative distribution of h
< SEL_DOM_SD_NEG >		SD for the negative distribution of h
< PROP_POS_SEL_DOM >		Mixture weight of the positive distribution of h
< PROP_SEL_LOCAL >		Proportion of selected sites that are only under selection in the sub-population where they arose.<br>(Only used if `-mg` is in command line)

Table 2: Tags that can appear in the `-p` parameter file.

This file specifies mutation and selection parameters, and parameters that control some details of the simulation run. See `Example/data/par_example.xml` for an example. Table 2 briefly describes the tags. To implement selection, the minimal FREGENE command is

```
fregene -SELECT -i infile1 -p infile2 -recomb infile3 -o outfile
```

The fitness (W) of an individual is obtained by summation over non-neutral SNPs:

$$W_i = 1 + \sum_j x_j \tag{1}$$

where

$$x_j = \begin{cases} 0 & \text{if the individual is an ancestral homozygote at site } j \\ sh & \text{if heterozygote} \\ s & \text{if derived homozygote.} \end{cases}$$

When a mutation occurs, it is under selection with probability $< PROP\_SEL >$. The intensity coefficient s (identified as `*_COEF_*` in the parameter file) and dominance coefficient h (referred as `*_DOM_*`) are each sampled as a mixture of two Gaussian distributions. For convenience, the first of these distributions is called "positive" (labelled `*_POS`) and the second is called "negative" (`*_NEG`), but their values need not reflect these labels. The user specifies the relative weight (between 0 and 1) of the positive distribution (`PROP_POS_SEL*`). If = 1, the negative distribution parameters are ignored; if = 0, the positive distribution parameters are ignored.

When a new selected site arises in a subdivided population, with probability `PROP_SEL_LOCAL` it is under selection only in the subpopulation where it arose. Otherwise, the site is under selection in all subpopulations.

Finally, each selected site is "switched off" (*i.e.* its selection and dominance coefficients are set to 0) with a probability specified by the `-sel_LE option` (Table 4). This is intended to allow the user to avoid accumulation of large numbers of sites under balancing selection, and also allows an equilibrium to be reached even when balancing selection is present. At each generation, a selected site is switched off with default probability 1/75,000 (corresponding to a mean time under selection of 75,000 generations if neither allele reaches fixation).

3.2 Recombination parameters (`-recomb file_name`; required)

The recombination model is hierarchical, and is highly flexible, allowing a uniform recombination rate, or rates that can vary both on a fine scale (hotspots) and on a broad scale.

Chromosomes are divided into `N_REGIONS` equal-size regions, each of which is subdivided into `SUBS_PER_REGION` equal-size subregions. The mean per-site recombination rate within a region is initially sampled from a Gamma distribution, with scale and shape parameters `REGION_GAMMA_SCALE` and `REGION_GAMMA_SHAPE`. However, the realised values are normalised so that the overall mean recombination rate is equal to `RECOM_RATE`. Thus, if there is only one region, its recombination rate is equal to `RECOM_RATE` irrespective of the parameters of the Gamma distribution. (NB in our parameterisation, the Gamma distribution with scale parameter α and shape parameter β has mean $\alpha\beta$ and variance $\alpha\beta^2$.)

Similarly, in each subregion the recombination rate is sampled from a Gamma distribution, but in this case there is no normalising. The shape parameter is specified by the user

**Variable Name** *(Default, if applicable)*	**Description**
<*GC_RATE*> *(0)*	Rate of gene conversion (GC) start sites (/bp/gener)
<*GC_LENGTH*> *(500)*	GC tract length (bp)
<*RECOM_RATE*> (10^{-8})	Average crossover (CO) rate (/bp/gener)
<*N_REGIONS*> *(1)*	Number of regions in each chromosome
<*SUBS_PER_REGION*> *(1)*	Number of sub-regions per region.
<*REGION_GAMMA_SCALE*> *(1)*<br><*REGION_GAMMA_SHAPE*> *(1)*	Scale and shape parameter of the Gamma distribution used to determine the rate for each region.
<*SUB_REGION_GAMMA_SHAPE*> *(1)*	Shape parameter of the Gamma distribution for rates within each sub-region (scale defined by the overall rate in the region).
<*PROP_RECOM_HS*> *(0)*	Proportion of CO occurring in hotspots
<*HS_LENGTH*> *(200)*	Length of CO hotspots (bp)
<*HS_SPACING*> *(5000)*	Average distance between HS (bp)
<*HS_SPACING_GAMMA_SHAPE*> *(1)*	Shape parameter of the Gamma distribution for distance between HS.
<*INTENSITY_GAMMA_SHAPE*> *(1)*	Shape parameter of the Gamma distribution for additional CO intensity within HS
<*HS_COMB*> *(0)*	1 if GC start sites have the same distribution as CO<br>0 if GC start sites are sampled uniformly.

Table 3: Tags that can appear in the recombination file.

(`SUB_REGION_GAMMA_SHAPE`), but the scale parameter is fixed by FREGENE so that the mean equals the region mean rate. Within each subregion, hotspots of fixed length (`HS_LENGTH`) are sampled such that the distance between hotspots follows a Gamma distribution with mean `HS_SPACING` and shape parameter `HS_SPACING_GAMMA_SHAPE`. From the mean recombination rate for the subregion, and the proportion of recombinations that occur in hotspots (`PROP_RECOM_HS`), FREGENE computes a background rate that applies to all sites as well as a mean rate within hotspots. The excess rate above background in a particular hotspot is sampled from a Gamma distribution with variance defined by its shape parameter (`INTENSITY_GAMMA_SHAPE`).

The start sites of gene conversions, with tract length `GC_LENGTH`, can be sampled uniformly (`HF_COMB=0`), or in proportion to crossover rates (`HF_COMB=1`) but with overall rate specified by `GC_RATE`.

4 Output files

4.1 Log file

When FREGENE is executing, summary information describing the initial population, the simulation features and the evolution of the population over generations are sent to standard output, and can be redirected to a log file (see `Example/log_example.xml`).

4.2 Main output file (`-o file_name`; required)

The output file is formatted to enable its reuse as a `-i` input file. It contains the basic information as presented in Section 3.1. This file also contains variables required to ensure that, during iterated runs, FREGENE works with appropriate values. These variables detailed below and summarized in Table 1.

- $< IS\_SCALED >$: $= 1$ if the output population is scaled. Then the output population size is the value specified by $< SEGMENTS >$ divided by the scaling factor;
  $= 0$ if the output population is unscaled: either the run was unscaled or a scaled run was terminated with a population expansion (using the `-sel_exp` option).

- $< SCALING\_FACTOR >$: (we refer to this as λ) If $\lambda \neq 1$, the assigned population size and number of generations for the simulation are both divided by λ, while the rate parameters are all multiplied by λ. This allows the target population to be approximated with reduced computation time and memory requirements.

- $< GROUPS >$ and $< GROUPS\_SIZE >$: gives the number of subpopulations and their respective sizes. These variables can be specified in the command line.

- $< SEED >$: the random seed is updated automatically in case of successive runs of FREGENE. However, to allow run replications, the user might want to specify the same seed for multiple runs. This variable can be defined in the command line.

- $< NB\_SWAPPED\_SITES >$ and $< LIST\_SWAPPED\_SITES >$: respectively the number and the location of sites for which the derived allele has become the major allele.

- $< NB\_SELECTED\_SITES >$: the number of sites under selection in the final generation. Each selected site is associated with a position ($< POSN\_SELECTED\_SITES >$), a generation at which it arose ($< SEL\_GENERATION >$), a value for the selection coefficient s and for the dominance coefficient h ($< SEL\_COEF >$ and $< SEL\_DOM >$), and the subpopulation(s). $< GROUP\_SELECTED\_SITES >$ specifies in which subpopulation(s) each site is under selection. If a site is under selection only locally (*i.e.* in the subpopulation where it arose), the number of the corresponding subpopulation is reported in that list. Otherwise, the site is under selection in all subpopulations, which is coded by `100` in the list.

4.3 Other output files enabled by additional options

Other files can be generated if the corresponding option, from the following list, is selected by the user in the command line:

- `-sel file_name` option:
  Calling this option, will print in `file_name` all the selected sites that have gone to fixation since the first generation (see `Example/data/sel_example.txt`). Selected sites are described by:

  1. Their position (`Posn`)
  2. Their s value (`SelCoef`)
  3. Their h value (`SelDom`)
  4. The generation at which they arose (`SelGeneration`)
  5. The generation at which the site reached fixation (`Gener`)
  6. The number subpopulation(s) in which the site is under selection: (`GroupSel`).

- `-hz file_name` option:
  If chosen, this option will print in `file_name` the homozygozity after each deletion interval (see `Example/data/homozgyosity_example.txt`). (N.B. We use "homozygosity" as shorthand for the probability that two random chromosomes are the same at a randomly-chosen site.)

- `-freq file_name` option:
  The allele counts of all polymorphic sites (under selection or not) in the final generation are printed in `file_name`. The first column is the position of the polymorphism and the second column is the corresponding allele count (see `Example/data/freq_example.txt`).

- `-ADDS file_name1 ...file_name6` option:
  This option requires five file names as argument:

  1. `file_name1` (see `Example/data/Monitor_example.txt`):
     In this file, each column corresponds to a generation. The first line gives the population mean fitness, the second and third lines are, respectively, the number of recombinant and non-recombinant individuals.
  2. `file_name2` (see `Example/data/List_fixed_example.txt`):
     This lists all selected sites that have gone to fixation. Each site is described as previously (see `-sel option`).
  3. `file_name3` (see `Example/data/List_deleted_example.txt`):
     The list of selected sites that have disappeared from the population.
  4. `file_name4` (see `Example/data/List_selected_example.txt`):
     The list of all sites that were under selection at any time in the the simulation.
  5. `file_name5` (see `Example/data/List_Kept_example.txt`):
     Sites that were under selection during at least two deletion operations.

6. `file_name6` (see `Example/data/List_SwitchedOff_example.txt`):
   Selected sites that were switched off during the simulation.

The use of this option is useful to follow up the mutations that have appeared in the population over generations as well as for describing the behavior of the selection model, but it can generate large files.

- `-af file_name` option:
  This option writes in `file_name` information about all polymorphic sites present at the last generation (see `Example/data/sim_example.txt`). For each site (column), it records the position (1st line); the population MAF (2nd line) and swapping status (3rd line). If a site is not swapped (=0), then the minor allele is the derived allele, if (=1) the minor allele is ancestral.

- `-bckMut file_name` and `-dbleHit file_name` options:
  These two options, which can be called separately, keep track of new mutations that arose at a polymorphic site. If the ancestral allele mutates, it is a double hit, whereas if it is the derived allele, the site reverts to the ancestral allele: a back mutation.

  Each double hit and/or back mutation are described as follows:

  – `Gener`: the generation at which the event occurred.
  – `Chromosome`: the number of the chromosome on which the event occurred.
  – `Posn`: the position on the chromosome.
  – `Swapped`: specifies if the sites where the event has been recorded was swapped when it was notified.
  – `Count`: gives the number of copies of the allele in the population, when the event occurred. If swapped, this number represents the number of copies of the derived allele, otherwise, this represents the number of derived copies that present in the population at the time of the event.

  Multi hit (triple or over), which may be important in case of high mutation rates (*e.g.* in viruses) are also tracked: if the same back mutation and/or double hit (*i.e.* same location, chromosome and generation) appears more than once in the file. This option requires alleles to be counted in each generation, which slows the algorithm.

- `-histAF int file_name` option:
  This option enables the distribution of allele frequency to be tracked. Calling this function will create a vector whose first element is the cumulative number of monomorphic sites (allele frequency =0), and whose next 100 elements are then the cumulative number of sites for all percentiles of the allele frequency. This vector is refreshed and printed out every `int` generations, and the resulting matrix is saved in `file_name`.

- `-RecOut file_name` option:
  If chosen this option will save in `file_name` a summary of the recombination model. Regions (`Level=0`), subregions (`Level=1`) and hotspots (`Level=2`) are first described by their beginning and ending position (`Posn_init` and `Posn_final`). Per-base recombination rates (`Intensity`) are also stored: for subregions, background rates over the whole subregion are reported and, for hotspots, this column corresponds to the per-base recombination rate applicable throughout the given hotspot.

5 Additional command line options

- `-SELECT` option:
  This option does not require any argument. It is used to enable selection in the model. If not used, some selection-specific steps of the algorithm are avoided and FREGENE runs quicker.

- `-SELFING float` option:
  If chosen, self-reproduction is enabled. One parent is sampled at random, and then the second parent is the same as the first with the probability specified, and is otherwise chosen at random among individuals other than the first parent.

- `-mg` option:
  This option has no argument and, if chosen, enables migration between subpopulations, according to the backward migration matrix (*.i.e.* the transpose of the migration matrix) defined in the parameter file.

- `-gn int` option:
  This option specifies the number of generations for the simulation run. If this option is chosen, the number of generations in the parameter file will be ignored.

- `-sub int int ... int` option:
  This option specifies the subpopulation structure of the starting population. Its first argument is the number of subpopulations (K). Then follows K arguments corresponding the initial population sizes (numbers of chromosomes) of each subpopulation. If subpopulation sizes are not consistent with the ones in the input file (*e.g.* in the case of successive runs), the run will terminate.

- `-subout int int ... int` option:
  This option allows the user to specify different sub-population sizes for the present run from those specified in the input file, arising from a previous run of FREGENE. The population expands (or contracts) in the first generation to the desired sizes. The number

Option USAGE	**Description**
`-i file_name`	Specifies the input file (required)
`-p file_name`	Specifies the parameter file (required)
`-recomb file_name`	Specifies the recombination model (required)
`-o file_name`	Specifies the output file (required)
`-SELECT void`	Enables selection
`-SELFING float`	Enables self reproduction with specified selfing fraction
`-mg void`	Enables migration between subpopulations
`-gn int`	Specifies the number of generations
`-sub int...int`	Describes the population structure: number and sizes of subpopulations
`-subout int...int`	Modifies the subpopulation sizes (but not the number of subpopulations)
`-os int...int`	Defines the number of sequences output in each subpopulation
`-r float`	Specifies the population growth rate, r (default 0 corresponds to no growth)<br>If $r \neq 0$, in each generation the (real-valued) population size (individuals)<br>is multiplied by $1+r$ then (temporarily) rounded up to an integer
`-sd int`	Specifies the random seed
`-scale float`	Enables scaling and specifies the scaling factor λ
`-scale_exp float int`	Like `-scale float` but adds extra generations (2nd argument) during<br>which the population expands linearly to reach the target population size<br>(specified by $< SEGMENTS >$).
`-sel file_name`	Location for list of selected sites gone to fixation
`-hz file_name`	Location for output of homozygozity over generations
`-freq file_name`	Location for counts of all minor alleles (selected or not) in final generation
`-ADDS file_1...file_6`	Locations for 6 tables used for detailed monitoring of simulation.
`-af file_name`	Location for description of polymorphic sites in final generation
`-bckMut file_name`	Location for list of back mutations
`-dbleHit file_name`	Location for list of double hits
`-histAF int file_name`	Specifies how often and where to save distribution of allele frequencies
`-RecOut file_name`	Location for positions and intensities of hotspots
`-sel_LE int`	Specifies the average # generations selected sites remain under selection<br>if neither fixed nor lost.

Table 4: Summary of FREGENE command line options. Further details are given in the text.

of subpopulations (first argument) must be equal to the one in the input file (or the one defined in the `-sub` option), otherwise FREGENE stops.

- `-os int int ... int` option:
  This option specifies the number of sequences (scaled) at output in each subpopulation. As for `-subout` option, the first argument (the number of subpopulations) must be consistent with the input file and/or `-sub` option. Furthermore, the number of sequences in each subpopulation must be lower or equal to the number of sequences simulated, otherwise, the run is stopped.

- `-r float` option:
  The population growth rate for subdivided populations (*i.e.* `-sub` option must also be assigned). It can also be set in the parameter file (see Table 2). Default value is 0, corresponding to a constant size population.

- `-sd` option:
  Specifies the random seed used. FREGENE automatically changes the seed between two successive runs, but setting the seed can allow replicate runs.

- `-scale float` option:
  This option enables the scaling of the population and its argument defines the scaling factor (λ). Scaling reduces by a factor λ both the population size and the number of generations, and increases similarly the overall recombination rate, the mutation rate and the selection coefficient s. The use of scaling results in a large drop in computation time and memory requirement at the cost of some approximation; in particular scaling increases the rate of double-hit and back mutations. The final population size will be λ times smaller than specified by $< SEGMENTS >$ in the input file.

- `-scale_exp float int` option:
  This option also enables the scaling of the simulation, but still outputs the number of chromosomes specified by $< SEGMENTS >$. A number of generations (2nd argument), is added to the simulation, during which the population expands linearly (the population size in each generation rounded up to an even integer).

- `-sel_LE int` option:
  The mean number of generations before selection is "switched off" at a selected site (default 75,000). The inverse of this number is the probability per generation that selection is switched off (so selection operates at each selected site for a time that is random with the geometric distribution, mean `LE`).

6 Using SAMPLE

SAMPLE generates (multiple) haplotype or genotype samples from a FREGENE output population. Either a case–control sample, random sample with a continuous phenotype or a random sample with no phenotype can be generated. The number of cases must be set zero if a continuous phenotype or random sample is required. Otherwise, cases/control status is assigned according to a disease model that is multiplicative over genotypes at each causal SNP, and also multiplicative over SNPs if the number causal SNPs selected is greater than 1. To generate individuals with a continuous phenotype the `-sigma` option must be selected, this parameter specifies the phenotypic standard deviation. The heritability of each SNP must be specified from which the regression coefficient are calculated;

$$\beta = \sqrt{\frac{\sigma^2 h^2}{2f(1-f)(1-h^2)}}$$

where f is the MAF, h^2 is the user specified heritability and the genotypes are coded as (-1, 0, 1). Thus, each individuals phenotype is sampled from the following Gaussian distribution

$$x \sim \mathrm{N}\left(\sum \beta_i x_i, \sigma^2\right)$$

where the x_i's are the genotypes at the selected causal SNPs. Causal SNPs are selected at random within allele frequency bands specified by the `-f` option.

All options are set on the command line.

- `-i file_name` **(required)**:
  Input file, usually the output file of a FREGENE run (*e.g.* `data/rin_example.xml`).

- `-og file_name` option:
  Records genotypes of sampled individuals. Case control status is first entry on each line, followed by genotypes coded as 0, 1, 2.

- `-oh file_name` option:
  Records haplotypes of sampled individuals in `file_name`, each haplotype on a separate line. For both haplotypes of an individual, the 1st column is its case/control status then alleles are recorded as 0 (major) or 1 (minor). Either `og` or `oh` must be specified; if both are specified then both output files are generated.

- `-scan file_name` option:
  File containing list of locations of SNPs output to the genotype and/or haplotype files.

- `-min int` option:
  Specifies the minimum MAF of loci that are output. If neither this option or `-scan` are selected all polymorphisms are written to file the genotype and/or haplotype files.

- `-chromolength float` option:
  This option takes as an argument a chromosome length in Mb; the returned chromosomes are then restricted to be of this length.

- `-LD file_name` option:
  Argument specifying output file that lists all pairs of SNPs within 200kb of each other and their pairwise r^2 value. (see `./data_sample/LD_example.txt`).

- `-sd int` option:
  Seed for random number generator.

- `-controls int` option:
  Number of controls to sample.

- `-samples int` option:
  Specifies the number of case-control, or just control, samples, default = 1.

To generate a phenotype, either binary or continous, the following options must be specified

- `-cases int` option, only required for a binary phenotype:
  This options specifies that the phenotype is binary and sets the number of cases to sample, default = 0.

- `-sigma float` option, only required for a continuous phenotype:
  This options specifies that the phenotype is continuous and sets the phenotypic standard deviation.

- `-prev float` option, only required for a binary phenotype:
  Disease prevalence, value between 0 and 1.

- `-snps int` option:
  Number of causal SNPs (*i.e.* that influence phenotype), default = 0.

- `-length int` option:
  Size (in Mb) of the chromosome the user wants to generate haplotype data for. If this option is not chosen, this chromosome length considered is equal to the one in FREGENE output file.

- `-f` $\texttt{float}_1$ `...` $\texttt{float}_{2k}$ option:
  Two arguments per causal SNP, specifying its minimum and maximum allele frequencies.

- `-rr` $\texttt{float}_1$ `...` $\texttt{float}_k$ option:
  One argument per causal SNP, if binary phenotype is specified the option specifies the risk ratio for both the heterozygote relative to the common homozygote, and also the

rare homozygote relative to the heterozygote. If a continuous phenotype is specified the option specifies the heritability attributed to each SNP. SNP order should be the same as for `-f`.

- `-log file_name` **(required)**:
  Records disease model details of each sampled file (see `data_sample/log_sample_example*.dat`).

7 Quick start guide

This section lists the main options of FREGENE. Although they are presented separately, they can be combined together. Running these lines in the `Example` directory, will only generate standard output but further outputs are available (see section 4.3).

- **To run a single-subpopulation simulation without selection**:
  `../Fregene_source/fregene -i data/in_example.xml -p data/par_example.xml`
  `-recomb data/recomb_example.xml -o data/rin_example.xml`

- **To run a selection model, use the `-SELECT` option**, and define selection parameters in `-p parameter file`.
  `../Fregene_source/fregene -i data/in_example.xml -p data/par_example.xml`
  `-recomb data/recomb_example.xml -o data/rin_example.xml -SELECT`

- **To consider subpopulation structure in the population, use the `-sub` option**, followed by the number of subpopulations and their size (here 2 subpopulations containing 1000, 4000 sequences, respectively):
  `../Fregene_source/fregene -i data/in_example.xml -p data/par_example.xml`
  `-recomb data/recomb_example.xml -o data/rin_example.xml -sub 2 1000 4000`.

- **To allow migration between subpopulations use the `-tt` option**. Migration rates are specified in the `-p parameter file`:
  `../Fregene_source/fregene -i data/in_example.xml -p data/par_example.xml`
  `-recomb data/recomb_example.xml -o data/rin_example.xml -sub 2 1000 4000 -mg`.

- **To consider population growth use the `-r` option**. The growth rate (here 1% per generation) is given as the argument of the option:
  `../Fregene_source/fregene -i data/in_example.xml -p data/par_example.xml`
  `-recomb data/recomb_example.xml -o data/rin_example.xml -r 0.01`.

- **To run a scaled simulation use the `-scale` option**, this dramatically speeds the program up (here the scaling factor λ is 10):
  `../Fregene_source/fregene -i data/in_example.xml -p data/par_example.xml`
  `-recomb data/recomb_example.xml -o data/rin_example.xml -scale 10.0`
  This will leave the user with 10 times fewer sequences in the output file. **To output the desired number of sequences , use the `-scale_exp` option**, where the first argument

is λ (here = 10) and the second argument is the number of extra generations during which population expands to reach the desired size (here, 100 generations):
`../Fregene_source/fregene -i data/in_example.xml -p data/par_example.xml -recomb data/recomb_example.xml -o data/rin_example.xml -scale_exp 10.0 100`

- **To enable self-reproduction, use the `-SELFING` option**. The proportion of individuals coming from a single parent, the selfing fraction, (here 10%) is the argument of the option:
`../Fregene_source/fregene -i data/in_example.xml -p data/par_example.xml -recomb data/recomb_example.xml -o data/rin_example.xml -SELFING 0.1`

Below is an example of a more sophisticated simulation that implies the use of `-sub`, `-subout` and, `-os` options.

- **Step 1: getting the initial panmictic population**:
The population size, specified in the input file, is 2,000 sequences. `../Fregene_source/fregene -i data/in_example.xml -p data/par_example1.xml -recomb data/recomb_example.xml -o data/rin_example.xml`

- **Step 2: Split in the population**:
2 subpopulations of 1,500 and 500 sequences are defined:
`../Fregene_source/fregene -i data/rin_example.xml -p data/par_example_2.xml -recomb data/recomb_example.xml -o data/rin_2_example.xml -sub 2 1500 500`
Use `-mg` option if the population are not independent.

- **Step 3: Extract the two subpopulations from the output**:
To achieve this, set the number of generations to 0 in `par_example_3.xml`.
For the first subpopulation, run:
`../Fregene_source/fregene -i data/rin_2_example.xml -p data/par_example_3.xml -recomb data/recomb_example.xml -o data/rin_Group1_example.xml -os 2 1500 0`
And for the second subpopulation:
`../Fregene_source/fregene -i data/rin_2_example.xml -p data/par_example_3.xml -recomb data/recomb_example.xml -o data/rin_Group2_example.xml -os 2 0 500`

- **Step 4: Working on each subpopulation separately**:
To enable the allele counts, the swapping and the selected sites to be updated on each subpopulations, a final step is required. The number of generations for this step can be 0, if the user is interested by the subpopulation at the end of step 2. Extra generations are possible if the user wants each subpopulation to evolve independently:
For the first subpopulation:
`../Fregene_source/fregene -i data/rin_Group1_example.xml -p data/par_example_4.xml -recomb data/recomb_example.xml -o data/rin_final_Group1_example.xml -sub 1 1500`

And for the second subpopulation:

```
../Fregene_source/fregene -i data/rin_Group2_example.xml -p data/par_example_4.xml
-recomb data/recomb_example.xml -o data/rin_final_Group2_example.xml -os 2 0 500
```
