# Supplementary material for: Fregene: Simulation of realistic sequence-level data in populations and ascertained samples
Source: BMC Bioinformatics. 2008 Sep 8;9:364. doi: 10.1186/1471-2105-9-364 (PMC2542380; doi:10.1186/1471-2105-9-364)
Supplement: Additional file 2 — FREGENE recombination model in detail. this text describes in greater details the recombination model implemented in FREGENE. [file 1471-2105-9-364-S2.pdf]

FREGENE **recombination model in detail**

The recombination model is based on a hierarchical approach where three levels are considered: sequences are divided into equal-size regions (first level) and subregions (second level) whose number is defined by the user. Then, within each subregion, a variable number of hotspots (third level) is located given the user-defined hotspot length and the parameters of the Gamma distribution from which the distance between hotspots is sampled.

For all three levels, a variable recombination intensity is sampled from a Gamma distribution with a variance defined by the user through its shape parameter, and which can be different for regions, subregions and/or hotspots. The mean of the distribution from which intensities for each region (green horizontal lines on figure 1) are sampled is defined by the per base per generation recombination rate entered by the user. For the two lower levels (subregions and hotspots), intensities are sampled such that, the mean intensity for the given level is consistent with the overall intensity of the higher level (respectively regions and subregions).

Each subregion is also associated with a 'background' rate (blue lines on figure 1), representing the base recombination rate for any site within the subregion (whether it belongs to a hotspot or not). Its computation involves the proportion of recombination occurring in hotspots, the user specifies. Finally, the height of each hotspot (red lines on figure 1) is obtained by adding to the background rate an extra intensity which is sampled from a Gamma distribution with mean depending on the overall intensity of the given hotspot and variance specified by the user.

In practice, for each gamete, the number of a recombination events (crossovers and gene conversions) is sampled from a Poisson distribution with mean proportional to the per-base recombination rate. Gene conversions can either be sampled uniformly along the sequence or located using the same 'recombination map' as the one designed for crossovers. In a first step, each recombination event is allocated both a region and a subregion with a probability proportional to the region and subregion intensities. Then within the sampled subregion the recombination is located either anywhere in the subregion (with probability proportional to the background rate of the subregion) or in one of the hotspots of the subregion (with probability proportional to the corresponding hotspot height).

This model can be simplified by reducing the number of level of the model (*e.g.* setting the number of regions and/or subregions to 1). It can be simplified even further by setting all variances to 0, which corresponds to a uniform recombination rate all along the sequence.
